# Characterizing differential individual response to porcine reproductive and respiratory syndrome virus infection through statistical and functional analysis of gene expression

**DOI:** 10.3389/fgene.2012.00321

**Published:** 2013-01-16

**Authors:** Maria E. Arceo, Catherine W. Ernst, Joan K. Lunney, Igseo Choi, Nancy E. Raney, Tinghua Huang, Christopher K. Tuggle, R. R. R. Rowland, Juan P. Steibel

**Affiliations:** ^1^Department of Animal Science, Michigan State UniversityEast Lansing, MI, USA; ^2^Animal Parasitic Diseases Laboratory, United States Department of Agriculture, Agriculture Research ServiceBeltsville, MD, USA; ^3^Department of Animal Science, Iowa State UniversityAmes, IA, USA; ^4^Department of Diagnostic Medicine and Pathobiology, Kansas State UniversityManhattan, KS, USA; ^5^Department of Fisheries and Wildlife, Michigan State UniversityEast Lansing, MI, USA

**Keywords:** porcine reproductive and respiratory syndrome, microarray, quantitative PCR, functional analysis, power analysis

## Abstract

We evaluated differences in gene expression in pigs from the Porcine Reproductive and Respiratory Syndrome (PRRS) Host Genetics Consortium initiative showing a range of responses to PRRS virus infection. Pigs were allocated into four phenotypic groups according to their serum viral level and weight gain. RNA obtained from blood at 0, 4, 7, 11, 14, 28, and 42 days post-infection (DPI) was hybridized to the 70-mer 20K Pigoligoarray. We used a blocked reference design for the microarray experiment. This allowed us to account for individual biological variation in gene expression, and to assess baseline effects before infection (0 DPI). Additionally, this design has the flexibility of incorporating future data for differential expression analysis. We focused on evaluating transcripts showing significant interaction of weight gain and serum viral level. We identified 491 significant comparisons [false discovery rate (FDR) = 10%] across all DPI and phenotypic groups. We corroborated the overall trend in direction and level of expression (measured as fold change) at 4 DPI using qPCR (*r* = 0.91, *p* ≤ 0.0007). At 4 and 7 DPI, network and functional analyses were performed to assess if immune related gene sets were enriched for genes differentially expressed (DE) across four phenotypic groups. We identified cell death function as being significantly associated (FDR ≤ 5%) with several networks enriched for DE transcripts. We found the genes interferon-alpha 1(*IFNA1*), major histocompatibility complex, class II, DQ alpha 1 (*SLA-DQA1*), and major histocompatibility complex, class II, DR alpha (*SLA-DRA*) to be DE (*p* ≤ 0.05) between phenotypic groups. Finally, we performed a power analysis to estimate sample size and sampling time-points for future experiments. We concluded the best scenario for investigation of early response to PRRSV infection consists of sampling at 0, 4, and 7 DPI using about 30 pigs per phenotypic group.

## Introduction

Porcine Reproductive and Respiratory Syndrome (PRRS) was initially described in the US over 20 years ago (Done et al., 1996). Overall, the disease causes $664 million annual losses to the US pork industry (Holtkamp et al., 2012). A virus, now known as Porcine Reproductive and Respiratory Syndrome Virus (PRRSV), has been identified as the primary causative agent (Collins et al., 1992). Viral replication takes place in the host's immune cells (Rowland et al., 2003; Genini et al., 2008) thereby, reducing the cytokine-mediated inflammatory response. While the molecular pathways involved in the protection against PRRS have not yet been entirely elucidated (Kimman et al., 2009), phenotypic variation between breed-lines has been observed in disease-related and production traits of experimentally infected pigs (Petry et al., 2005; Vincent et al., 2006; Doeschl-Wilson et al., 2009). These authors reported differences in clinical symptoms and lung pathology in response to PRRSV infection, as well as in virus levels in serum and/or respiratory tissues, such as lung and bronchial lymph nodes. Doeschl-Wilson et al. (2009) and Petry et al. (2005) also reported differential body weight changes in PRRSV infected pigs. A possible way of reducing PRRS incidence would be to breed pigs that are more resistant to the disease. To this end, host genetic response to infection can be studied using currently available genomic tools (Lewis et al., 2007; Lunney and Chen, 2010).

Studying gene expression in pigs showing phenotypic variation to PRRSV infection responses will enhance our knowledge of genetic control of the susceptibility to this disease. In this context, differential expression of a reduced number of immune related genes has been evaluated (Petry et al., 2007; Lunney et al., 2010) and global differential expression has been assessed *in vivo* (Bates et al., 2008; Xiao et al., 2010a,b; Zhou et al., 2011; Wysocki et al., 2012) and *in vitro* (Lee et al., 2004a,b; Miller and Fox, 2004; Genini et al., 2008; Ait-Ali et al., 2011).

Most previous studies focused on comparing gene expression of PRRSV-infected and uninfected pigs, as well as gene expression between animals showing differences in post-infection viral titers. However, little is known of the interaction between viral load (VL) and weight gain as it relates to gene expression post-infection. This is particularly important given the reported associations of immune traits with growth rate (Galina-Pantoja et al., 2006; Boddicker et al., 2012) and the genetic correlations between growth rate and disease traits (Doeschl-Wilson et al., 2009) as well as between growth rate and immune related traits (Clapperton et al., 2009).

Furthermore, most previous studies assessed differential expression of specific virus target tissues or cells. In addition, different researchers have addressed the ability of the blood transcriptome to reflect the transcriptome of other body tissues in humans (Liew et al., 2006; Mohr and Liew, 2007; Kohane and Valtchinov, 2012). In our system, identifying differential gene expression in whole blood in response to PRRSV infection would facilitate genome testing and diagnosis of suceptibility to the disease.

The availability of whole genome microarrays (Steibel et al., 2009c) and next generation sequencing (Mardis, 2008) have further favored whole genome expression profiling of PRRSV infected animals (Xiao et al., 2010a,b). Important features when evaluating gene expression are: (1) the correct modeling of the phenotypic variation and the inclusion of biological replication (Rosa et al., 2005) and (2) sampling relevant tissues and time-points (Mateu and Diaz, 2008; Lunney et al., 2010).

We evaluated whole-genome expression profile of pigs assigned to four reaction groups (phenotypic groups) according to the pigs' weight gain and VL as part of the PRRS Host Genetics Consortium (PHGC) (Lunney et al., 2011). The goals of this study were: (1) to assess global differential gene expression in whole blood of commercial pigs showing variation in phenotypic response to PRRSV experimental infection, and to identify relevant molecular networks and biological functions enriched for differentially expressed (DE) genes involved in the pig's immune response to PRRSV infection; and (2) to inform the design of future experiments, to determine the most informative early time-points and sample sizes required for powerful inferences when assessing gene expression in blood of commercial pigs experimentally infected with PRRSV.

## Materials and methods

### Animal model and study design

Crossbred commercial pigs (~200) from PHGC trial one (Lunney et al., 2011) were transported to the Kansas State University bio-secure testing facility at weaning (11–21 days old) and allocated to pens (10–15 pigs/pen). Pigs came from PRRSV-, Influenza virus- and *Mycoplasma hyopneumoniae*-free farms. After a 7-days acclimation period and antibiotic treatments, pigs were both intramuscularly and intranasally infected with a known isolate of PRRSV (10^5^ tissue culture infectious dose_50_ of NVSL 97-7985). Blood samples were collected in Tempus™ Blood RNA Tubes (Life Technologies, Carlsbad, CA) at 0, 4, 7, 11, 14, 19, 28, 35, and 42 days post-infection (DPI). We followed the manufacturer's standard RNA purification protocol including DNase treatment to obtain blood RNA and remove any remaining genomic DNA. Serum viral level was quantified using a semi-quantitative TaqMan® PCR assay. Individual animal weight was measured at weekly intervals. More details on the pig resources, study design and data storage are in Lunney et al. (2011) and Boddicker et al. (2012). The study was approved by Kansas State University Institutional Animal Care and Use Committee.

### Phenotypic groups

**Figure 1:**
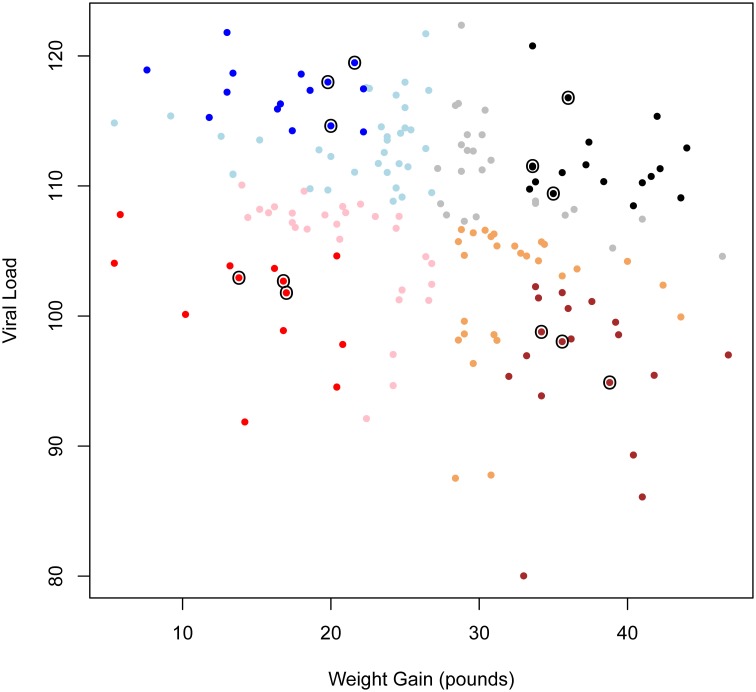
**Scatterplot of weight gain vs. viral load for all pigs in PHGC trial one.** Each dot represents a pig. Color shadings indicate the four different phenotpypic groups (HvHg, HvLg, LvHg, and LvLg). Dark color indicates pigs that were classified into one of the groups. Light color indicates pigs that were not classified because they lay in the boundary of the groups. Circles indicate pigs that were selected for transcriptional profiling in this experiment.

Figure 1 shows a scatterplot of weight gain vs. VL for all pigs from PHGC trial one. Four phenotypic groups were defined according to the pigs' weight gain and VL. Weight gain was defined as body weight (kg) from 0 to 42 DPI. VL was defined as the area under the curve of the log-transformed serum viral level from 0 to 21 DPI. The choice of time-points to define VL was based on observations of viral levels over time for 565 pigs across three infection trials (Boddicker et al., 2012). These results showed that the majority of challenged pigs had peak viremia from 4 to 21 DPI (Lunney et al., 2011). Viremia past 21 DPI was not taken into account because the viral levels rebounded in ~33% of pigs (Boddicker et al., 2012). Since for these infection trials pigs were followed until 42 DPI it was most advantageous to follow weight gain for the more extended time (42 DPI). The two variables (VL and weight gain) showed moderate negative correlation (*r* = −0.29). Thus, bivariate data of VL and weight gain were centered at their mean values and rotated to obtain uncorrelated measures. Phenotypic groups were then specified as a combination of these two traits: (1) high VL-high weight gain (HvHg), (2) high VL-low weight gain (HvLg), (3) low VL-high weight gain (LvHg), and (4) low VL-low weight gain (LvLg). For allocation to these four groups, pigs that were within one standard deviation of the population mean for either of the traits was discarded and the remaining animals were classified to one of the groups (Figure 1).

### Microarray design and analysis

Three pigs per group were randomly selected and their RNA isolated using the Tempus™ Spin RNA Isolation Kit as per manufacturer's instructions (Applied Biosystems/Life Technologies) from blood at 0, 4, 7, 11, 14, 28, and 42 DPI. RNA samples were reverse transcribed using the Amino Allyl MessageAmp II aRNA Amplification Kit (Ambion./Life Technologies), labeled with N-hydroxysuccinate (NHS) ester Cy3 or Cy5 dyes (GE Healthcare, CA), and hybridized to the 20K 70-mer oligonucleotide Pigoligoarray as previously described (Steibel et al., 2009c) following a block reference design (Steibel and Rosa, 2005). Each individual pig's 0-DPI-sample served as reference for all other samples from the same animal. Reference sample dye flipping was performed across pigs to allow separation of dye and 0-DPI effects (Steibel and Rosa, 2005). Fluorescent images and fluorescence intensity data were collected as previously described (Steibel et al., 2009c). Median intensities were background corrected with Normexp method fixing the offset parameter κ = 50 (Ritchie et al., 2007). Background corrected data was normalized using a within print-tip loess-location normalization (Yang et al., 2002). All computations were implemented in R (R Development Core Team, 2010) through LIMMA (Smyth, 2005). Normalized log-intensities were analyzed on a transcript per transcript basis using a linear mixed model accounting for all pertinent random and fixed effects (Rosa et al., 2005) as described below:

$$y_{ijklm}=\mu +TG_{ij}+D_{l}+A_{m}+S_{k}+e_{ijklm}$$

where *y*_*ijklm*_ is the log-intensity measure at the *i*^*th*^ DPI, for the *k*^*th*^ pig corresponding to phenotypic group *j* in the *m*^*th*^ array labeled with the *l*^*th*^ dye; μ is the overall mean; *TG*_*ij*_ is the effect of DPI *i* and phenotypic group *j* in the expression of the transcript, with *i* = 1, …, 7 and *j* = 1, …, 4; *D*_*l*_ is the effect of *l*^*th*^ dye, with *l* = 1, 2; and *A*_*m*_ is the random effect of the *m*^*th*^ array, with *m* = 1, …, 72 and *A*_*m*_ ~ *N*(0, σ^2^_*a*_); *S*_*k*_ is the random effect of the *k*^*th*^ pig, with *k* = 1, …, 12 and *S*_*k*_ ~ *N*(0, σ^2^_*s*_); finally *e*_*ijklm*_ is the residual with *e*_*ijklm*_ ~ *N*(0, σ^2^_*e*_).

The mixed model was fitted using the package MAANOVA (Wu et al., 2003). An *F*-test based on a shrinkage estimator of variance components was used to evaluate significance of fixed effects (Cui et al., 2005). Permutation based *p*-values (number of permutations = 100) were obtained to assess significance (Yang and Churchill, 2006).

To account for multiple testing a two-stage testing procedure was used to assess significance of gene expression changes in response to weight gain and VL status over time. First, for each DPI, the interaction effect *TG*_*ij*_ was tested at 10% false discovery rate (FDR) (Storey, 2003). Then, for all transcripts with significant interaction, the effect of VL was evaluated in high-weight-gain and low-weight-gain pigs, separately (nominal *p* ≤ 1/Ns, with Ns = number of significant interactions). Similarly, the effect of weight gain was evaluated in high-viral-load and low-viral-load pigs, separately.

All in all, this testing protocol resulted in 4 contrasts of interest for the comparisons of phenotypic groups. Each contrast involved the comparison of two phenotypic groups. Two of these contrasts evaluated the effect of VL and the other two the effect of weight gain. In particular, for the VL effect, we compared (1) HvHg vs. LvHg and (2) HvLg vs. LvLg. To evaluate the weight gain effect, we compared (1) HvHg vs. HvLg and (2) LvHg vs. LvLg.

Data have been deposited in NCBI's Gene Expression Omnibus (GEO) (http://www.ncbi.nlm.nih.gov/geo/) with accession number GSE41144. Code used for these analyses can be found at https://www.msu.edu/~steibelj/JP_files/PRRSV.html

### Pathway analysis

Gene set enrichment analyses were performed using Ingenuity Pathways Analysis software (IPA; Ingenuity® Systems, www.ingenuity.com). Annotation for the oligonucleotides present in the microarray is available from http://www.animalgenome.org/cgi-bin/host/Lunney/oligoAnnotatn. Details have been published in Steibel et al. (2009c). After statistical analyses described above pathway analyses were restricted to early time-points (4 and 7 DPI). The network analyses generated a set of relevant networks (*p* ≤ 10^−10^ and number of DE genes ≥ 5) built based on a user-specified list of genes. Networks were composed of genes and gene products that are known to interact with each other and that were enriched for the DE transcripts (as defined in section “Microarray design and analysis”). Functional analysis identified the biological functions that were significantly enriched [Benjamini and Hochberg (1995) *p* < 0.05] for these DE genes.

### qPCR design and analysis

Quantitative PCR (qPCR) analysis was used to assess differential expression of 15 genes. Twelve genes were selected from microarray and pathway analyses results. In addition, three genes [interferon-alpha 1 (*IFNA1*); major histocompatibility complex, class II, DQ alpha 1 (*SLA-DQA1*); and major histocompatibility complex, class II, DR alpha (*SLA-DRA*)] not present in the microarray platform were included based on previously documented knowledge of their relevant role in immune modulation, reviewed by Lunney and Chen (2010). Probes and primers were obtained from the Porcine Immunology and Nutrition Database (Dawson et al., 2005) or designed with Primer Express Software v3.0 (Life Technologies) from sequences obtained from Ensembl (http://useast.ensembl.org/index.html). Primers and probes were designed to span exon-exon junctions whenever possible. Sequences for all primers and probes are provided in Table A1. Synthesis of cDNA was performed with SuperScript Reverse Transcriptase® and qPCR amplification was implemented using the Brilliant Kit (Agilent Technologies, Inc., CA) with 35 ng of cDNA in an ABI Prism 7500 Sequence Detector System (Life Technologies). Assays were performed in duplicate. The amplification conditions are described in Royaee et al. (2004). *Ct* values were obtained from each individual amplification curve. Average *Ct* for each target gene in each sample and DPI (4 and 7) were subtracted from the corresponding average *Ct* of *RPL32* (housekeeping gene), producing Δ *Ct* values. ΔΔ *Ct* values were computed by subtracting 0-DPI-Δ *Ct* from Δ *Ct* at each DPI. Resulting Δ Δ *Ct* were analyzed separately for each DPI (except 0 DPI) with the following linear model:

$$y_{m}=G_{m}+e_{m}$$

where *y*_*m*_ is the Δ Δ *Ct* value for the gene in the *m*^*th*^ phenotypic group, *G*_*m*_ is the effect of the phenotypic group *m* and *e*_*m*_ ~ *N*(0, σ^2^_*e*_) is the residual. This model is equivalent to a previously described linear model (Steibel et al., 2009a).

### Statistical power and sample size computation

Using this experiment's dataset as pilot data for future experiments with a similar design, we computed the expected discovery rate (EDR) and FDR as defined by Gadbury et al. (2004) to estimate statistical power at a fixed number of biological replicates (*n*) and type I error rate (α). The EDR is the multi-test equivalent to power, which is also called sensitivity (Steibel et al., 2009b). EDR should be computed at a specific nominal type I error rate, α, and for a given sample size, conditioning on estimated effects from a previous experiment (Gadbury et al., 2004). We considered either *n* = 20 or *n* = 30 per phenotypic group (four groups) and α = 0.01. This choice of α resulted in a FDR <10% in all cases. Computations were performed using PowerAtlas software (Page et al., 2006). Sample sizes were selected assuming a common reference design with either 4 (*n* = 20) or 3 (*n* = 30) sampling time-points, such that the total number of microarray slides was fixed to 240. This represents a common situation where the researcher has to decide whether to allocate arrays to extra biological samples with fewer time-points or to include more time-points at the expense of sample size for a given total budget.

## Results

Dye labeled cDNA prepared from blood samples from 12 PHGC pigs at seven different time points (0, 4, 7, 11, 14, 28, and 42 DPI) were hybridized to the Pigoligoarray using a block reference design. Three pigs per group were randomly selected from each of the four phenotypic groups defined according to the pigs' weight gain and VL (HvHg, HvLg, LvHg, and LvLg). We addressed global differential expression in four contrasts of interest (HvHg vs. LvHg, HvLg vs. LvLg, HvHg vs. HvLg, and LvHg vs. LvLg).

### Microarray analysis

#### Evidence of differential gene expression at 0 DPI

**Figure 2:**
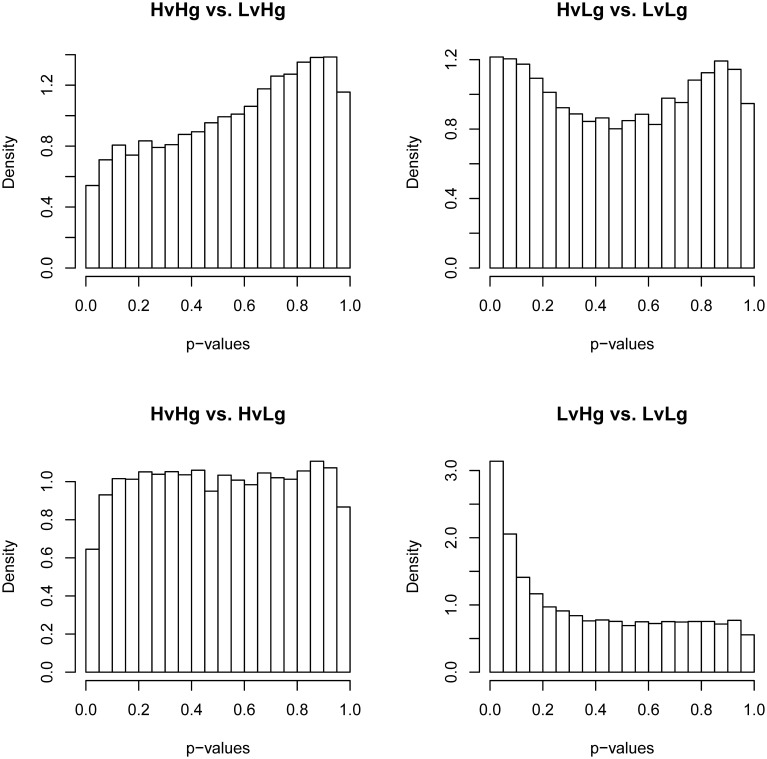
**Histogram of *p*-values for the four contrasts of interest at 0 DPI.** For each contrast of interest (HvHg vs. LvHg, HvLg vs. LvLg, HvHg vs. HvLg, and LvHg vs. LvLg), this figure shows the distribution of *p*-values at 0 DPI. For a condition of no differential expression the histograms should have a flat trend.

The presence of an effect on gene expression profile that cannot be attributed to the experimental infection was addressed by evaluating differential gene expression between the four phenotypic groups at 0 DPI. Although no significant differences in transcripts were identified (FDR ≤ 0.1), inspection of *p*-value distributions for the four contrasts indicated a departure from the expected uniform distribution under null hypothesis (Figure 2). The actual distribution of *p*-values for LvHg vs. LvLg indicated an excess of small *p*-values. This is consistent with the alternative hypothesis of differential expression. The contrasts HvHg vs. LvHg and HvLg vs. LvLg showed *p*-value distributions inconsistent with both null and alternative hypotheses implicit in the analysis model (Page et al., 2006). The observed deviations in the *p*-value distributions of these tests likely reveal the existence of unaccounted effects (Page et al., 2003). These patterns also appeared in contrasts at other time-points if these differences at 0 DPI were ignored (results not shown).

#### Evidence of differential gene expression for remaining DPI

Based on the results from the previous section, differential expression between phenotypic groups after 0 DPI was corrected by subtracting the estimated difference at 0 DPI. For example, to address differential expression between two phenotypic groups of pigs, the effect was estimated following [(*TG*_*i* ≠ 0, *j*_ − *TG*_*i*_′ = 0, *j*) − (*TG*_*i* ≠ 0, *j*′_ − *TG*_*i*′ = 0, *j*′_)]. The same procedure was used for all contrasts. After correcting for 0-DPI estimated effect, the distribution of *p*-values for all contrasts was consistent with the expected distribution under either null or alternative hypotheses (data not shown). This indicated that correcting each comparison estimate by the corresponding estimate at time zero accounts for pre-existing differences in gene expression and/or for animal specific effects missed by the model. Consequently, we based all inferences on the above specified contrasts.

**Figure 3:**
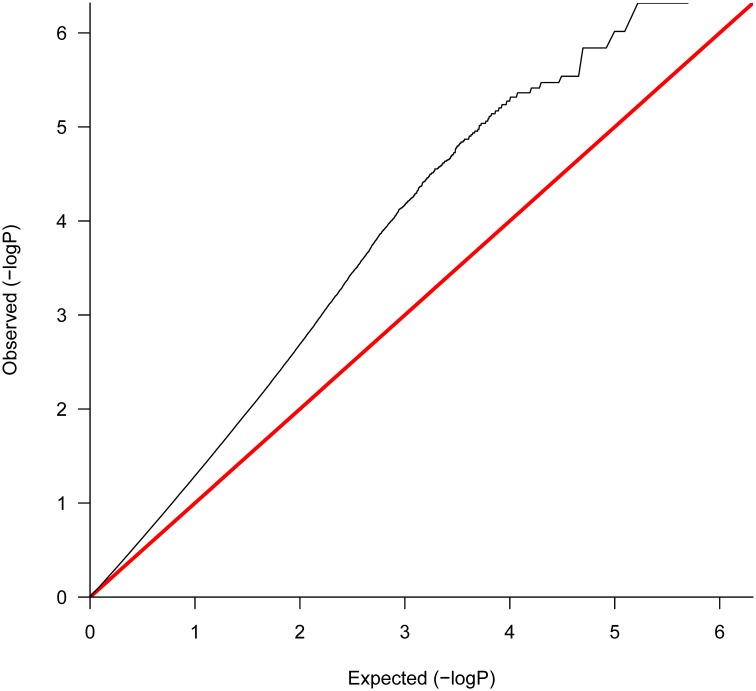
**Uniform Q-Q plot for gene expression of all contrasts across 4–42 DPI after correcting for 0 DPI estimated effect.** This plot represents the quantiles of the empirical distribution of *p*-values vs. the expected quantiles of uniformly distributed *p*-values (corresponding to the null hypothesis). The represented departure from the straight line *y* = *x* indicates an excess of small *p*-values as compared to the expectation under the null hypothesis, consistent with the alternative hypothesis of differential expression.

Evidence of differential expression was found, as revealed by the q – q plot of *p*-values (Figure 3). This plot represents the quantiles of the empirical distribution of *p*-values vs. the expected quantiles of uniformly distributed *p*-values (corresponding to the null hypothesis). The represented departure from the straight line *y* = *x* indicates an excess of small *p*-values as compared to the expectation under the null hypothesis, consistent with the alternative hypothesis.

#### Weight gain and viral load interaction effect on gene expression

The number of transcripts showing a significant interaction was 288, 14, 177, and 12 at 4, 7, 14, and 42 DPI respectively (Table 1). There were no significant interactions detected at 11 and 28 DPI. Transcripts showing significant weight gain and VL interaction effect on their expression were further evaluated and results are presented in section “Weight gain and viral load effect on gene expression.”

**Table 1:** **Number of putatively differentially expressed transcripts**.

**Time (DPI)**	***N*s**	**Phenotypic groups comparisons**			
		**HvHg vs. LvHg**	**HvLg vs. LvLg**	**HvHg vs. HvLg**	**LvLg vs. LvHg**
4	288	86	42	22	141
7	14	13	12	14	11
14	177	106	25	38	120
42	12	12	12	9	12

Numbers indicate differentially expressed genes per time and phenotypic groups' comparisons.

Ns, total number of transcripts being tested (i.e., with a significant interaction at the specific DPI being evaluated).

#### Weight gain and viral load effect on gene expression

To declare a DE transcript we used an adjusted *p*-value where the null hypothesis was rejected if *p* ≤ 1/Ns, with Ns = total number of transcripts being tested (i.e., with a significant interaction at the specific DPI being evaluated), such that we expected one false positive per comparison. This led to a number of putatively DE transcripts per DPI and comparison, as shown in Table 1. At 4 and 14 DPI, we observed a similar number of DE transcripts, consisting of a large number of transcripts with significant interaction (288 and 177 respectively) mainly involving differential expression in HvHg vs. LvHg (86 and 106 transcripts) and in LvLg vs. LvHg (141 and 120 transcripts). However, at 7 and 14 DPI, the number of significant interactions is smaller (14 and 12) with most of the transcripts DE across all four contrasts. Although we reported the number of DE transcripts at 42 DPI, we did not follow up on these results because responses at that late sampling time-point could be due to a rebound of the viral replication (Boddicker et al., 2012).

### Pathway analyses

Subsequent to testing for changes in global gene expression, we assessed if immune related gene sets were enriched for DE genes across pigs from the four phenotypic groups. We performed pathway analyses to identify relevant molecular networks and biological functions associated with such networks enriched for the DE genes identified in section “Weight gain and viral load effect on gene expression.” We restricted the analyses to 4 and 7 DPI since (1) we were interested in early immune responses and (2) these were the times that provided the most power to detect future differential expression (as shown in section “Microarray statistical power and sample size estimation”). Following these pathway analyses, and to limit the interpretation of results to genes with large effects, in addition to the *p*-value threshold described in section “Weight gain and viral load effect on gene expression,” we considered an absolute fold-change (FC) threshold equal to, or greater than 1.5. For instance, in a specific contrast involving two groups, the gene expression level in the first phenotypic group had to be 50% larger (or smaller) than the one in the second phenotypic group for a significantly DE gene to be considered. DE genes with a positive FC (larger than 1.5) were considered to be over-expressed, and DE genes with a negative FC (smaller than −1.5) were considered to be under-expressed in the first phenotypic group included in the contrast equation.

Several gene networks enriched for DE genes were recognized for the four contrasts of interest. The significant functional categories identified in these networks, as well as selected DE genes associated with these categories are listed in Table 2. Cell Death function was significantly identified at 4 and 7 DPI. At 4 DPI we identified major histocompatibility complex, class II, DR beta 1 (*HLA-DRB1/SLA-DRB1)* involved in the activity of Th1 cells, which was under-expressed in the HvLg vs. LvLg contrast. At 7 DPI, genes involved in the initiation of apoptosis (PYD and CARD domain containing, *PYCARD*) and cytotoxicity of T cells (Granzyme A, *GZMA*) were under-expressed in the HvHg vs. LvHg and HvHg vs. HvLg contrasts. PYCARD was over-expressed and associated with Genetic Disorder and Imflammatory disease in HvLg vs. LvLg. GZMA was also associated with Cell Morphology in the LvHg vs. LvLg contrast and with Cellular compromise in HvLg vs. LvLg, where it was over-expressed.

**Table 2:** **Functional categories enriched for DE genes at 4 and 7 DPI**.

**Contrast**	**4 DPI**		**7 DPI**	
	**Functional categories**	**Genes (1)**	**Functional categories**	**Genes (1)**
HvHg vs. LvHg	Cell death, cell morphology, cellular assembly and organization, cellular function, and maintenance (5^*^)	c-mer proto-oncogene tyrosine kinase, *MERTK* (−); Ezrin, *EZR* (+); Moesin, *MSN* (−)	Cell death (1^*^)	PYD and CARD domain containing, *PYCARD* (−); Granzyme A, *GZMA* (−)
HvLg vs. LvLg	Organismal development, cell death (3^*^)	Major histocompatibility complex, class II, DR beta 1, *HLA-DRB1/SLA-DRB1* (−); Jumonji AT rich interactive domain, *JARID2* (−)	Genetic disorder, inflammatory disease, cellular compromise (1^*^)	PYD and CARD domain containing, *PYCARD* (+); Granzyme A, *GZMA* (+)
HvHg vs. HvLg	None identified (1^*^)		Cell death (1^*^)	PYD and CARD domain containing, *PYCARD* (−); Granzyme A, *GZMA* (−)
Hg vs. LvLg	None identified (7^*^)		Cell morphology (1^*^)	Granzyme A, *GZMA* (+)

Functional categories (FDR < 5%) associated with significant networks (p ≤ 10^−10^ and number of DE genes ≥ 5).

(1) Selected DE genes associated with functional categories at 4 DPI (adjusted p-value ≤ 0.0035) and 7 DPI (adjusted p-value ≤ 0.07).

(+) Over-expressed in the first phenotypic group in the contrast.

(−) Under-expressed in the first phenotypic group in the contrast.

* Number of significant networks identified in the specific contrast and DPI.

### qPCR analysis

#### Verification of microarray findings

Among a total of 96 comparisons for the 12 genes present in the microarray and selected for qPCR (12 genes × 4 phenotypic groups × 2 DPI), 26 significant comparisons (*p* ≤ 1/Ns, as defined in section “Weight gain and viral load effect on gene expression”) were detected by the microarray and two were confirmed by the qPCR. Significant comparisons occurred for nine genes: *EPS15*, *EZR*, *GRLF1*, *GZMA*, *ITGB7*, *JARID2*, *MERTK*, *PYCARD*, and *RASGRP1*. The qPCR confirmed differential expression of two genes: *JARID2* and *ITGB7*. *JARID2* was confirmed in HvLg vs. LvLg at 4 DPI (*p* ≤ 0.03 and *p* ≤ 0.0001 for QPCR and microarray, respectively). *ITGB7* was confirmed in LvHg vs. LvLg at 7 DPI (*p* ≤ 0.007 and *p* ≤ 0.004). We also evaluated the gene set correlations between microarray and qPCR measured FC. From a measurement error perspective, the overall trend in direction and amount of expression was validated at 4 DPI (*r* = 0.91, *p* ≤ 0.0007), but not at 7 DPI.

These results indicate that, although the rate of differential expression validation of individual genes is limited, the overall pattern of differential expression was confirmed for comparisons at 4 DPI. Consequently, enrichment analysis (networks and functions) identified at the earliest time-point are expected to be reproduced in future experiments.

#### Additional genes

Three genes not present on the microarray were also tested using qPCR: *IFNA1*, major histocompatibility complex, class II, DQ alpha 1 (*HLA-DQA1*/*SLA-DQA1*), and major histocompatibility complex, class II, DR alpha (*HLA-DRA*/*SLA-DRA*). SLA class II antigens are expressed in B and T cells, with numerous haplotypes identified throughout different pig populations, which led researchers to explore their association with disease responses (Lunney et al., 2009). *IFNA1* encodes for an innate cytokine, and has been reported to be modulated by PRRSV (Mateu and Diaz, 2008; Kimman et al., 2009). Significance levels and FC for these genes in all comparisons are presented in Table 3.

**Table 3:** **Test of immune gene expression for genes not present in the microarray platform**.

**DPI**	**Symbol**	**Viral comparisons**				**Growth comparisons**			
		**HvHg vs. LvHg**		**HvLg vs. LvLg**		**HvHg vs. HvLg**		**LvHg vs. LvLg**	
		***p*-value**	**FC**	***p*-value**	**FC**	***p*-value**	**FC**	***p*-value**	**FC**
4	IFNA	0.09	3.21	**0.01**	**−6.67**	**0.02**	**5.45**	**0.05**	**−3.93**
	SLA-DQA	0.71	1.15	0.11	−1.92	0.71	1.15	0.11	−1.92
	SLA-DRA	0.96	−1.03	0.62	−1.35	0.71	−1.25	0.42	−1.64
7	IFNA	**0.04**	**3.25**	**0.02**	**−4.08**	**0.04**	**3.27**	**0.02**	**−4.05**
	SLA-DQA	**0.04**	**1.32**	**0.01**	**−1.43**	0.95	−1.01	**0.00**	**−1.89**
	SLA-DRA	0.15	1.58	0.55	−1.20	0.40	−1.29	**0.02**	**−2.45**

Values in the table include significance levels and FC of genes; bolded values indicate significant comparisons and their FC.

At 4 DPI, *IFNA1* was significantly DE (*p* ≤ 0.05) when comparing HvLg–LvLg and HvHg–HvLg.

At 7 DPI, *IFNA1* was significantly DE in all contrasts. *SLA-DQA* was DE in all contrasts except for HvHg vs. HvLg and *SLA-DRA* was DE in LvHg vs. LvLg.

### Microarray statistical power and sample size estimation

In order to inform the design of future experiments, we computed the EDR per contrast at early DPI. We considered a future experiment that would include sampling time = 0 DPI plus two or three other times, selected among 4, 7, and 11 DPI. We assumed effect sizes estimated from this data, a fixed nominal error rate α = 0.01, and two sample sizes (*n* = 30 for three sampling time-points or *n* = 20 for four sampling time-points). The sample allocation (sampling fewer time points with more biological replicates or vice versa) would require the same number of microarray slides (240) and would roughly have the same cost. For evaluating which sampling time-points have to be included in a future study, we set the threshold of inclusion to EDR > 80%. That is, the average probability of detecting an effect (assuming the effect is indeed present) to be larger than 0.8. When *n* = 30, two sampling time-points (other than 0 DPI) could be included. In that case, the best sampling combination would be at 4 and 7 DPI. This would provide adequate power (EDR > 80%) to detect weight gain and VL effects in all contrasts except for LvHg vs. LvLg. Changing the sampling scheme to include 11 DPI (with only 20 samples per phenotypic group), would not add to the purpose of having sufficient power in that contrast. Furthermore, sampling at 11 DPI would only result in one contrast (HvLg vs. LvLg) having EDR > 80% (Table 4).

**Table 4:** **Expected discovery rate (EDR) comparing 20 and 30 biological replicates**.

**Phenotypic group comparisons**	**Sample size (*n*)**	**Sampling time-points (DPI**)		
		**4**	**7**	**11**
HvHg vs. LvHg	20	**0.84**	**0.86**	0.37
	30	**0.91**	**0.92**	0.47
HvLg vs. LvLg	20	**0.83**	0.80	**0.96**
	30	**0.91**	**0.88**	**0.98**
HvHg vs. HvLg	20	0.77	**0.88**	NA
	30	**0.86**	**0.94**	0.45
LvHg vs. LvLg	20	0.55	0.42	0.62
	30	0.63	0.51	0.70

All four contrasts were compared at each DPI and evaluated for future sampling with desirable power (>80%).

NA, not available. The algorithm could not reach a result.

## Discussion

The first objective of this study was to assess global differential gene expression in weaned pigs showing variation in weight gain and blood VL in response to PRRSV infection. To achieve this objective, four reaction groups (phenotypic groups) of pigs were evaluated. Our study is different from other PRRSV-response gene expression profiling experiments in three ways. First, we focused on modeling individual biological variation of gene expression. Given that a longer term objective is to find genes for diagnosis and prognosis of PRRSV infection, we were interested in characterizing the variance of expression at the individual level. The resulting scope of inference is different from that obtained using pooled samples (Genini et al., 2008; Xiao et al., 2010a,b), since our experimental and inferential unit is the individual animal and not a pool of animals. Second, we used a blocked reference design (Steibel and Rosa, 2005). In contrast to common reference designs (Bates et al., 2008; Xiao et al., 2010a,b; Wysocki et al., 2012), our design allowed us to use 0 DPI samples as a reference, and still include 0 DPI in tests, which was instrumental in assessing baseline effects before infection. Additionally, because of the design used to accommodate single cDNA samples, this study has the flexibility of incorporating future data for differential expression analysis. Third, we report on whole-genome expression profiling of blood cells from *in vivo* infected pigs that complements existing results from studies using pulmonary alveolar macrophages (PAM), bronchial lymph node and lung (Petry et al., 2007; Bates et al., 2008; Genini et al., 2008; Lunney et al., 2010; Xiao et al., 2010a; Zhou et al., 2011; Wysocki et al., 2012). Furthermore, obtaining blood samples is simpler and less invasive than sampling other tissues, thus simplifying implementation of genomic diagnostics in pigs, including *in situ* sampling at farms.

We first assessed differential expression at 0 DPI between pigs allocated to different phenotypic groups, and observed effects that could not be solely attributed to experimental infection or random errors (Page et al., 2006). The individual baseline (0 DPI) differential expression assessment was only briefly reported before (Ait-Ali et al., 2011), and using 0 DPI correction has not been reported. This type of correction was not usually addressed either because the baseline samples were pooled (Genini et al., 2008; Xiao et al., 2010a,b) or because they were omitted from the expression experiment (Bates et al., 2008; Wysocki et al., 2012). Differences in gene expression between phenotypic groups before infection could be due to the different genetic background of the individual pigs (Lunney and Chen, 2010). Within a species, individuals can vary greatly in their resistance to infections, and a major part of this variability may be attributed to the variation in the genetic background (Ardia et al., 2011). Several researchers have reported genetic variation in immune traits in healthy pigs (Clapperton et al., 2005, 2009; Flori et al., 2011). We believe our baseline assessment is of particular importance since this genetic background could influence the response to the disease. Consequently, accounting for pre-existing differential expression should be considered in future infection studies.

We tested the interaction effect between weight gain and VL on global gene expression. From breeding and management perspectives, quantifying interaction effects at early time-points would allow making timely management and selection decisions. Consequently we concentrated on 4–14 DPI for further analyses. In addition, by considering early DPI we assured that the effect being evaluated was exclusively due to an initial infection stage and not to a rebound of the disease (Boddicker et al., 2012). Our results complement and extend those reported by Petry et al. (2007) and Bates et al. (2008), who tested the interaction between viral burden and genetic line as well as infection status on gene expression. Petry et al. (2005) reported that pigs from Nebraska Index Line, selected for improved reproductive traits, gained more weight than Hampshire × Duroc crossbred pigs after PRRSV infection. Therefore, the weight gain and blood viral level interaction effect we evaluated resembled the genetic line by viral burden interaction reported by Petry et al. (2007) and Bates et al. (2008). These authors reported seven genes with significant line by VL interaction in lung or bronchial lymph node expression profiles. Querying expression levels for the same genes in our dataset, we found that *DDX3Y* (DEAD box proteins, ATP-dependent RNA helicase), a paralog of *DDX3* reported by Bates et al. (2008), was also DE in blood cells.

Significant differences in *DDX3* expression occurred between low and high viral burden pigs from their Nebraska Index Line, but not in Hampshire × Duroc line. Likewise, we observed significant differences (*p* ≤ 0.003) in *DDX3Y* expression in HvHg vs. LvHg, but not in HvLg vs. LvLg, at 14 DPI, although the direction of change was opposite in our results (over-expressed in HvHg pigs) as compared to the Bates et al. (2008) experiment. However, our results are in agreement with those from Genini et al. (2008) who reported up-regulation of a gene from the same family (DDX17) in PRRSV infected PAM. Moreover, DEAD-box helicases are involved in all aspects of RNA metabolism. Specifically, in humans DDX3 was reported to participate in innate immune signaling and to enhance anti-viral responses by promoting IFN production (Schroder, 2010; Ulvila et al., 2010). DDX3 was also reported as a target for viral manipulation (Schroder, 2010). Thus, the over-expression of DDX3 molecules in HvHg pigs could be attributed either to a host anti-viral response or to a viral mechanism for replication (Ulvila et al., 2010).

Within the first objective of this study we also aimed to characterize gene networks and individual genes influencing PRRSV immune response in the four phenotypic groups considered. We further evaluated transcripts with expression subject to significant VL by weight gain interaction effects to identify biological functions associated with relevant molecular networks. We focused on 4 and 7 DPI since these provide insights on early host anti-viral and innate immune response to PRRSV infection and they are candidate sampling time-points to be pursued in future studies. Pathway analysis revealed that cell death function was significantly associated with several networks enriched for DE genes at 4 and 7 DPI. Genes included in these networks and associated with cell death were *MERTK*, *GZMA*, and *PYCARD*. All these genes followed a general pattern of under-expression in high VL compared to low VL pigs (*FC* ≤ −1.5). An exception to this was HvLg vs. LvLg at 7 DPI. These overall results are consistent with those from Genini et al. (2008) that reported inhibition of apoptosis in cell lines 9–12 h post infection. Results obtained with our type 2 isolate of PRRS virus are also comparable to results obtained from piglets infected with a different and highly pathogenic type 2 strain of the virus (HP-PRRSV) comparing gene expression to uninfected controls at 4 and 7 DPI (Xiao et al., 2010b). Cell death is a host defense mechanism to inhibit viral replication (Alcami and Koszinowski, 2000). Overall, the global gene expression profile showed a trend where HvLg and HvHg pigs had lower expression of the listed genes relative to LvLg and LvHg pigs, respectively, indicating that the defense mechanism mediated by cell death had reduced efficiency, thereby allowing increased viral replication. At 4 DPI, our study identified *MERTK* as DE and associated with cell death in HvHg vs. LvHg pigs. The product of *MERTK* is a phagocytic receptor that is involved in the clearance of apoptotic thymocytes. Mouse macrophages lacking *MERTK* showed a delayed clearance of apoptotic cells (Seitz et al., 2007). There have been no previous reports of this gene identified as DE in PRRSV response studies.

Key genes in the swine leukocyte antigens (SLA) complex have been well documented for their effects on production and immune traits in different pig populations (Lunney et al., 2009). At 7 DPI, we identified *SLA-DRA* significantly over-expressed in LvLg relative to LvHg. *SLA-DQA1* followed the same trend, and in addition, it was significantly under-expressed in LvHg and HvLg relative to HvHg and LvLg, respectively. Global differential expression and functional analysis comparing PRRSV infected to uninfected pigs at the same sampling time-points by Xiao et al. (2010a) reported that MHC class II antigens (*SLA-DQA*, *SLA-DMB*, *SLA-DQB1*, and *SLA-DRA*) were significantly induced in PRRSV infected lungs.

Our study identified IFNA1 as being significantly DE in all contrasts but HvHg vs. LvHg at 4 DPI. Specifically, at both 4 and 7 DPI, *IFNA1* was over-expressed in HvHg and LvLg relative to HvLg and LvHg pigs, respectively. *IFNA* was reported as under-expressed in PRRSV-infected with respect to uninfected PAM at 30 (Ait-Ali et al., 2011) but not at 12 h post infection (Genini et al., 2008). *IFNA* was reported under-expressed at 4 and 7 DPI in lung tissue of infected pigs relative to uninfected controls (Xiao et al., 2010a) but at 14 DPI, Lunney et al. (2010) reported no differences in expression in tracheobronchial lymph node for several innate markers (*IFNA*, *IL1B*, and *IL8*). In addition, Petry et al. (2007) found that differences in expression of *IFNA* were influenced by pig genetic line.

Overall, our findings of DE genes in whole blood are in agreement with previous reports on specific target tissues and cells, such as lung and PAM, following PRRSV infections and provide evidence of the immune response against a pathogen. Changes in blood transcriptional profiles were reported in humans with different non-systemic infectious diseases (Chaussabel et al., 2010) and non-hematological disorders (Liew et al., 2006; Mohr and Liew, 2007). Comparison of the peripherial blood expressed transcripts with expressed transcripts of different solid tissues in humans, resulted in ~80% of shared transcripts, and in the postulation of this tissue as a surrogate tissue (Liew et al., 2006). More recently, Kohane and Valtchinov (2012) quantified and reported a high overlap between transcripts with the highest levels of expression in white blood cells and highly expressed transcripts in a mixture of other tissues. Therefore, evaluating the host blood transcriptome should provide useful diagnostic and/or disease prognosis information (Liew et al., 2006; Mohr and Liew, 2007; Chaussabel et al., 2010). Since blood cells interact with most body tissues, these cells reflect the state of other tissues (Kohane and Valtchinov, 2012). Our results stress the usefulness of our study for sampling the more accessible blood to reveal the complexity of host responses to PRRSV infection. We expect that our planned, more detailed studies will generate further answers on the role of these and many other genes in anti-PRRSV responses.

Finally, our global differential expression results were used as pilot data to inform design of future time-course transcription profiling experiments. We evaluated different scenarios of sample sizes and sampling time-points for combinations given a fixed total sampling effort. We concluded the best scenario for future studies consists of sampling at 4 and 7 DPI using about 30 pigs per phenotypic group, and that a minimum of 20 pigs per group are needed for controlling type I and type II error rates to acceptable levels in most comparisons. The results obtained with a sample size *n* = 30 were consistent with previous results obtained from a dataset generated by Chen et al. (manuscript in preparation). Our group used the Wysocki et al. (2012) dataset of lung tissue expression at 14 DPI to evaluate statistical power of high versus low viral burden pigs, and affirmed that approximately the same sample size was needed. These results underscore the importance of computing sample size. We predict that this could be applied in a broader context, for instance, in next generation sequencing experiments. Such technology is being increasingly used for evaluating expression profiling in pigs infected with PRRSV (Xiao et al., 2010a,b). Even though we could expect less technical variation in expression measured with RNA-seq (Marioni et al., 2008), biological variation would remain unaffected. In such cases, the only way of increasing power of the tests would be increasing the number of biological samples (Steibel et al., 2009b).

Evidence presented in this paper highlights the importance of thoughtful experimental design and accurate modeling. We acknowledge sample size is a key factor of every experiment and correct modeling of variation (biological and technical) is essential. As a result, this experiment provided information on actual sample sizes and sampling time-points needed for more precise estimation of effects of interest. Our preliminary results have already identified differential gene expression, molecular networks and biological functions affecting the four phenotypic groups of pigs and the influence of PRRSV infection. Finally, due to the flexible experimental design utilized in this study, the resulting dataset can be merged with future data for increasingly powerful and precise inferences on response to PRRSV infection.

### Conflict of interest statement

The authors declare that the research was conducted in the absence of any commercial or financial relationships that could be construed as a potential conflict of interest.

## Appendix

**Table A1:** **Primers and probes used to amplify genes evaluated with qPCR**.

**Gene**	**Ensembl accesion number**	**Primer foward**	**Primer reverse**	**Probe**
EPS15	ENSSSCT00000004284	AGCGAGGTTCAGGATCTTCAAG	GGGCCTTCTGCTCATCCA	CCCAGAAACAGCAAGTACAGGAACTCCTTG
EZR	ENSSSCT00000004487	GCAGCGGCAGCTGATGA	GTCGTTGTGGGTCCTCTTGTTC	ACCAACGAGCTGTCCCAGGCCA
GRLF1	ENSSSCT00000003446	CCCATACAACATGCAGATGGAT	ACCTCCTTTAGGGCATGTAGCTT	TGGAGGCACACAAAATCAACGACCG
GZMA	ENSSSCG00000016902	GGAGCTCACTCGATAACCAAGAAA	GCTTTAGAAGTTTAAGGTCACCCTCAT	TCCTTATCCATGCTTTGACCAGGACACAC
IFNA1	GQ415055 (GenBank)	TCAGCTGCAATGCCATCTG	AGGGAGAGATTCTCCTCATTTGTG	TGACCTGCCTCAGACCCACAGCC
IL1A	ENSSSCG00000008090	CTGAAGAAGAGACGGTTGAGTTTAAA	AAGTTGTATTTCATGTTGCTCTGGAA	CAGAAGAAGAAATCATCAAGCCCAGATCAGC
IL8	ENSSSCG00000008953	CCGTGTCAACATGACTTCCAA	GCCTCACAGAGAGCTGCAGAA	CTGTTGCCTTCTTGGCAGTTTTCCTGC
IRF1	ENSSSCG00000014277	AATCCAGCCCTGATACCTTCTCT	GGCCTGTTCAATGTCCAAGTC	TGCCTGATGACCACAGCAGCTACACA
ITGB7	ENSSSCG00000000257	TCGCAGCCCAGAGTTTGACT	GGCACTGGTGACAGCAAAGA	TCAGGTAGCCCAGGCCCTCTCTGC
JARID2	ENSSSCT00000001155	CCTTTCTCTGCCTTCGAGGTT	CGTCCTGAGAGCTTCCGAAAT	TCCTGCGCTGCCCAACAGCA
MERTK	ENSG00000153208	GGAAAGATGGGAAGGAATTGC	TCATCTTACAGATATATGACCCATTGTCT	TTCAGCATAACCAGTGTGCAGCGTTCA
PYCARD	ENSG00000103490	CAAACCAGCACTGCACTTCGT	CAGCCCGTCCACGTCTGT	CGGGCAGCCCTCATCTCAAGGG
RASGRP1	ENSSSCG00000004791	GGAGAATAAAGAATCCCTCATAAAATCA	TTATTTCCTGTTCCAGCTCTTGGT	CTCCGTCACCTCAGACTCCCCACC
RPL32	ENSSSCG00000027637	GGAAAGATGGGAAGGAATTGC	TCATCTTACAGATATATGACCCATTGTCT	TTCAGCATAACCAGTGTGCAGCGTTCA
SLA-DQA1	ENSSSCG00000001456	GGTTCCTGAGGTGACTGTGTTT	GACAGAGTGCCCGTTCTTCAA	CTGGGTCAGCCCAACACCCTCAT
SLA-DRA	ENSSSCG00000001453	CCCGCCAGTGGTCAATGT	AGTGGAACTTGCGGAAAAGG	AGGAGTGTCAGAGACAGTCTTCCTGCCC
